# Geospatial cluster analyses of pneumonia-associated hospitalisations among adults in New York City, 2010–2014

**DOI:** 10.1017/S0950268818003060

**Published:** 2018-11-19

**Authors:** P. A. Kache, T. Julien, R. E. Corrado, N. M. Vora, D. C. Daskalakis, J. K. Varma, D. E. Lucero

**Affiliations:** 1 Department of Epidemiology, Mailman School of Public Health, Columbia University Medical Center, New York City, USA; 2 New York City Department of Health and Mental Hygiene, New York City, USA; 3 Career Epidemiology Field Officer Program, Division of State and Local Readiness, Centers for Disease Control and Prevention (CDC), Atlanta, USA; 4 National Center for Emerging and Zoonotic Infectious Diseases, CDC, Atlanta, USA

**Keywords:** Hospitalisations, New York City, pneumonia

## Abstract

Pneumonia is a leading cause of death in New York City (NYC). We identified spatial clusters of pneumonia-associated hospitalisation for persons residing in NYC, aged ⩾18 years during 2010–2014. We detected pneumonia-associated hospitalisations using an all-payer inpatient dataset. Using geostatistical semivariogram modelling, local Moran's *I* cluster analyses and *χ*^2^ tests, we characterised differences between ‘hot spots’ and ‘cold spots’ for pneumonia-associated hospitalisations. During 2010–2014, there were 141 730 pneumonia-associated hospitalisations across 188 NYC neighbourhoods, of which 43.5% (*N* = 61 712) were sub-classified as severe. Hot spots of pneumonia-associated hospitalisation spanned 26 neighbourhoods in the Bronx, Manhattan and Staten Island, whereas cold spots were found in lower Manhattan and northeastern Queens. We identified hot spots of severe pneumonia-associated hospitalisation in the northern Bronx and the northern tip of Staten Island. For severe pneumonia-associated hospitalisations, hot-spot patients were of lower mean age and a greater proportion identified as non-Hispanic Black compared with cold spot patients; additionally, hot-spot patients had a longer hospital stay and a greater proportion experienced in-hospital death compared with cold-spot patients. Pneumonia prevention efforts within NYC should consider examining the reasons for higher rates in hot-spot neighbourhoods, and focus interventions towards the Bronx, northern Manhattan and Staten Island.

## Introduction

Pneumonia is a clinical syndrome characterised by infection of the lungs. Common aetiologic agents include *Streptococcus pneumoniae* and influenza virus, with clinical manifestations ranging from mild symptoms to severe illness and death [1]. Infections can occur within the community setting or in association with healthcare settings [2].

Together, ‘pneumonia and influenza’ rank as the third leading cause of death in New York City (NYC), with most deaths attributed to an underlying cause of pneumonia, not influenza [3]. During 2010–2014, 54.3% of pneumonia-associated hospitalisations among adults in NYC were due to community-acquired pneumonia (CAP), 30.2% to healthcare-associated pneumonia (HCAP), 14.0% to hospital-acquired pneumonia (HAP) and the remaining 1.6% to ventilator-associated pneumonia (VAP) [4]. While the distribution of each setting of acquisition is known to vary across the five boroughs of NYC, the distribution within each borough has not yet been assessed.

To develop pneumonia prevention strategies that consider the epidemiological variation within and between NYC boroughs, we sought to identify spatial clusters with significantly higher rates of pneumonia-associated hospitalisations for NYC residents aged ⩾18 years during 2010–2014. We also conducted cluster analyses of pneumonia-associated hospitalisations by severity and setting of acquisition.

## Methods

### Study site and population

Our study population consisted of persons residing in NYC, aged ⩾18 years who were admitted to a New York State (NYS) acute care facility with a pneumonia-associated hospitalisation during 1 January 2010–31 December 2014.

### Data source and definitions

The NYC Neighborhood Tabulation Area (NTA) was the geographic unit of analysis for this study. NTAs were created by the NYC Department of City Planning using aggregates of whole census tracts [5]. Based on the 2010 US Census, 2168 census tracts define the geopolitical sub-divisions of NYC, and correspond to 195 NTAs. The NTA provides a statistically reliable alternative to low population denominators and high sampling error associated with individual census tracts. A majority of NTAs within NYC are residential neighbourhoods (*N* = 188); however, several non-residential NTAs throughout the city include public domains such as parks, correctional facilities and airports (*N* = 7) [5]. Individuals housed within facilities (correctional, psychiatric, substance abuse treatment, etc.) located in non-residential NTAs were excluded from our study population. For this investigation, we used the NTA shapefile in a projected geographic coordinate system, New York Long Island FIPS 3104 North American Datum of 1983/Universal Transverse Mercator zone 18N (NAD83/UTM zone 18N).

Hospital discharge data were obtained through the Statewide Planning and Research Cooperative System (SPARCS) [6]. SPARCS is an all-payer reporting system, mandated to collect data on inpatient and outpatient hospital visits across NYS under Section 28.16 of the Public Health Law [7]. Data for each inpatient admission include patient demographics, geocoded address information, admission and discharge dates, International Classification of Diseases, Ninth edition, Clinical Modification (ICD-9-CM) principal and secondary diagnoses, and patient disposition upon discharge (e.g. whether in-hospital death occurred), among other variables. We analysed the most recent database available for each year (July 2014 release for years 2009 and 2010, June 2015 for 2011 and October 2015 for 2012–2014).

An ICD-9-CM principal diagnosis code indicates the condition chiefly responsible for a patient's admission to the hospital [8]. Within SPARCS, healthcare staff are able to specify up to 24 secondary diagnosis codes on a medical discharge record for conditions that either coexist at the time of patient admission, develop subsequent to hospitalisation, affect the patient's course of treatment or lengthen the hospital stay.

For this investigation, we defined a ‘non-severe pneumonia-associated hospitalisation’ as any inpatient discharge record having a principal diagnosis of pneumonia. A ‘severe pneumonia-associated hospitalisation’ was one with a principal diagnosis of sepsis or respiratory failure and a secondary diagnosis of pneumonia [9]. Taken together, we defined an ‘overall pneumonia-associated hospitalisation’ more broadly as one that could be non-severe or severe (Table 1). Hereafter we use the term ‘patient’ in reference to an individual who experienced a pneumonia-associated hospitalisation.

**Table 1.** ICD-9-CM classification of overall pneumonia-associated hospitalisation among adults in New York City, 2010–2014

Category		Principal diagnosis (ICD-9-CM^a^ code)	Secondary diagnosis (ICD-9-CM^a^ code)
Overall pneumonia-associated hospitalisation	Non-severe pneumonia-associated hospitalisation^b^	480–486, 487.0, 488.01, 488.11, or 488.81	–
	Severe pneumonia-associated hospitalisation^c^	518.81, 518.82, 518.84, 799.1, 038, 785.52, 995.91 or 995.92	480–486, 487.0, 488.01, 488.11 or 488.81

a International Classification of Diseases, Ninth Revision, Clinical Modification.

b Inpatient discharge record with a principal diagnosis of pneumonia.

c Inpatient discharge record with a principal diagnosis of sepsis or respiratory failure and a secondary diagnosis of pneumonia.

Setting of acquisition for a pneumonia-associated hospitalisation was classified as part of a previous study in accordance with Infectious Disease Society of America (IDSA) and American Thoracic Society (ATS) professional guidelines [2, 4].

NYC residency was based on a patient's home address within one of five NYC boroughs (Manhattan, Bronx, Brooklyn, Queens and Staten Island), which correspond to NYS counties (New York, Bronx, Kings, Queens and Richmond, respectively).

### Ethical considerations

This investigation involved analyses of existing deidentified hospitalisation data. The Centers for Disease Control and Prevention (CDC) determined this activity to be public health non-research, NYC Department of Health and Mental Hygiene (DOHMH) determined this activity exempt from federal regulations for the protection of human research subjects, and Columbia University Medical Center (CUMC) approved the protocol under expedited review.

### Analytical methods

#### Rates of pneumonia-associated hospitalisation

We calculated average annual, age-adjusted rates of overall pneumonia-associated hospitalisation for each residential NTA. Rates were calculated for each geographic unit, however not for each categorical age group. Annual age-stratified hospitalisation frequencies were normalised with DOHMH population estimates modified from 2010 Census denominators, and age-adjusted using the direct method, according to 2000 Census guidelines [10, 11]. For the direct method of standardisation, the number of hospitalisation events for each age group was first divided by the estimated population of each age group, then multiplied by a constant of 100 000 persons to calculate the age-specific hospitalisation rate. This age-specific rate was then multiplied by the proportion of the US standard population for the age group. Age-specific results were summed to calculate the age-adjusted hospitalisation rate [11].

Analogous calculations were conducted for severe pneumonia-associated hospitalisation, as well as for CAP, HCAP and HAP. We excluded analyses of VAP at the NTA-level, given the large number of non-zero counts less than 10, which may have poor reliability when converted to age-adjusted rates [12].

#### Geostatistical analyses

We developed a semivariogram model to first evaluate if NTA-level pneumonia-associated hospitalisation rates were significantly clustered. If the model demonstrated clustering, we calculated an average radius of clustering, referred to as the range of spatial autocorrelation. Spatial autocorrelation indicates if values for locations that are nearby to one another are more similar than values for locations that are more distant. If we were able to successfully fit a semivariogram model and define a range of spatial autocorrelation, we can assume that, within this average radius, NTAs that are nearby to one another have hospitalisation rates that are more similar than hospitalisation rates for NTAs that are more distant [13]. Fitting a semivariogram model required sequentially developing an empirical, experimental and statistical semivariogram (Electronic Supplementary Material, Methods) [14, 15].

We developed semivariogram models for rates of overall pneumonia-associated hospitalisation, as well as for each sub-classification of interest (severe pneumonia-associated hospitalisation rates, CAP, HCAP and HAP hospitalisation rates). Results were validated using the Incremental Spatial Autocorrelation (ISA) tool in ArcMap 10.2.1. This tool runs a global Moran's *I* analysis for increasing distances and provides a corresponding *z*-score to measure the intensity of spatial clustering. We compared the range of spatial autocorrelation from the semivariogram model to the first distance with a peak *z*-score, as calculated by the ISA tool. If results of the semivariogram model and ISA indicated clustering across NYC overall, we went on to assess local clustering, as described in the *Cluster analyses* section. The range of spatial autocorrelation was used as a fixed bandwidth parameter for spatial weighting within these cluster analyses.

#### Cluster analyses

To identify clusters, or neighbourhoods within close proximity of one another with similar pneumonia-associated hospitalisation rates, we conducted local Moran's *I* cluster analyses [16]. The geographical summation of local Moran's *I* relationships result in either statistically significant (1) clusters of nearby neighbourhoods with similarly high pneumonia-associated hospitalisation rates (hot spots); (2) clusters of nearby neighbourhoods with similarly low pneumonia-associated hospitalisation rates (cold spots); (3) neighbourhoods with high rates near low-rate neighbourhoods or neighbourhoods with low rates near high-rate neighbourhoods (spatial outliers); (4) or regions without statistically significant spatial clustering [16]. Statistical significance was estimated using 999 Monte Carlo simulations at a 5% level of significance. Additional details about geostatistical and cluster analyses are provided in Electronic Supplementary Material, Methods.

Finally, to better understand the profile of clusters for overall, severe and CAP-associated hospitalisation, we compared mean age using the Wilcoxon rank-sum test, and examined the median and interquartile range (IQR) for hospital length of stay (LOS). Additionally, we used *χ*^2^ tests to compare the proportion of patients in hot spots *vs.* cold spots according to sex, race/ethnicity, discharge location after hospitalisation and in-hospital death.

All statistical and spatial analyses were conducted using R 3.3.1 (R Core Team, Vienna, Austria) and ArcMap 10.2.1 (ESRI Inc., Redlands, CA, USA) [17].

## Results

### Rates of pneumonia-associated hospitalisation

During 2010–2014, there were 141 730 overall pneumonia-associated hospitalisations across 188 residential NTAs. Among these pneumonia-associated hospitalisations, 43.5% (*N* = 61 712) were sub-classified as severe pneumonia-associated hospitalisations. The majority of overall pneumonia-associated hospitalisations were caused by CAP (*N* = 88 420; 62.4%), followed by HCAP (*N* = 38 576; 27.2%), HAP (*N* = 12 292; 8.7%) and VAP (*N* = 2442; 1.7%).

**Fig. 1.**
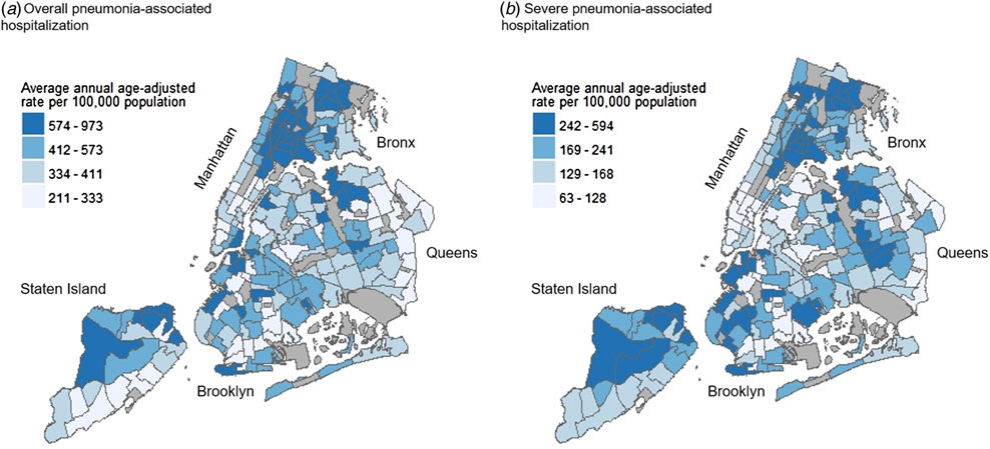
Average annual age-adjusted pneumonia-associated hospitalisation rates by severity among adults in New York City (NYC), 2010–2014. Maps showing NYC neighbourhoods according to average annual age-adjusted hospitalisation rates of overall pneumonia-associated hospitalisation (a) and severe pneumonia-associated hospitalisation (b). Labels indicate the five NYC boroughs (Manhattan, Bronx, Brooklyn, Queens and Staten Island). Hospitalisation rates were calculated for each residential Neighborhood Tabulation Area (NTA) based on hospital discharge data from the New York Statewide Planning and Research Cooperative System, and are divided into quartile classifications, with an equal number of residential NTAs in each class. Higher hospitalisation rates are shown in darker blue, and lower hospitalisation rates shown in lighter blue. Non-residential NTAs were excluded from the analysis and are shown in grey.

**Fig. 2.**
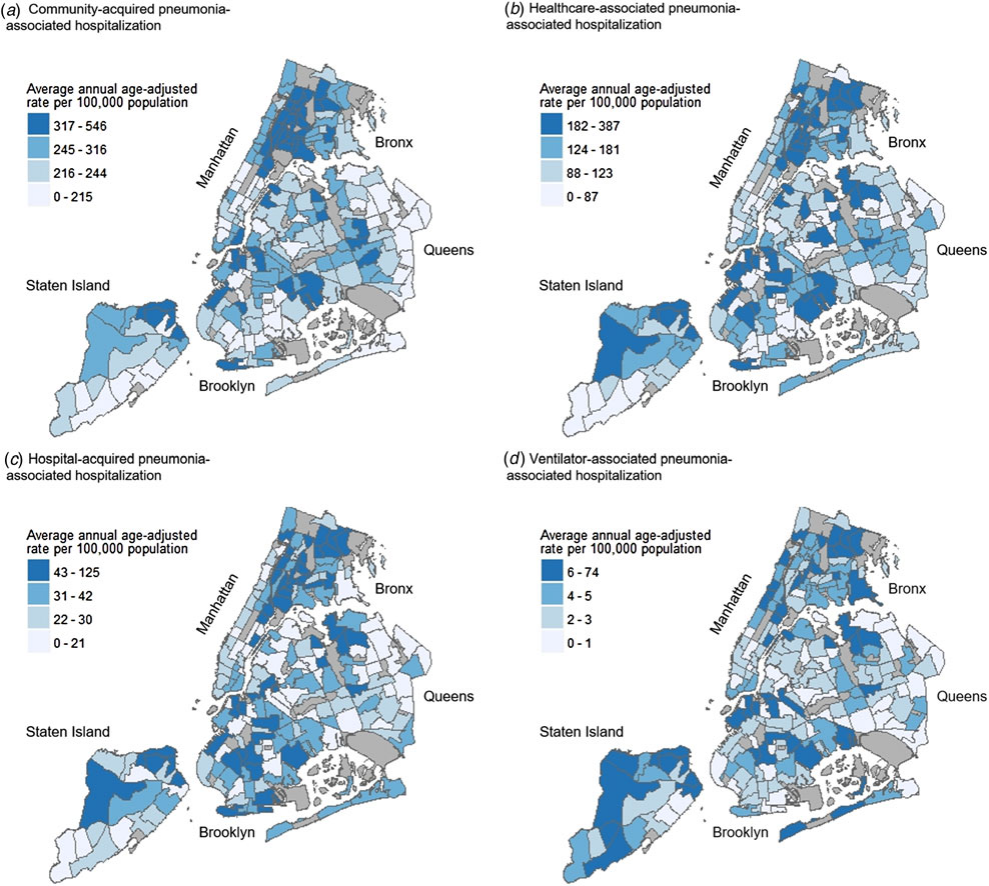
Average annual age-adjusted pneumonia-associated hospitalisation rates by setting of acquisition among adults in New York City (NYC), 2010–2014. Maps showing NYC neighbourhoods according to average annual age-adjusted hospitalisation rates of community-acquired pneumonia-associated hospitalisation (a), healthcare-associated pneumonia-associated hospitalisation (b), hospital-acquired pneumonia-associated hospitalisation (c), and ventilator-associated pneumonia-associated hospitalisation (d). Labels indicate the five NYC boroughs (Manhattan, Bronx, Brooklyn, Queens and Staten Island). Hospitalisation rates were calculated for each residential Neighborhood Tabulation Area (NTA) based on hospital discharge data from the New York Statewide Planning and Research Cooperative System, and are divided into quartile classifications, with an equal number of residential NTAs in each class. Higher hospitalisation rates are shown in darker blue, and lower hospitalisation rates shown in lighter blue. Non-residential NTAs were excluded from the analysis and are shown in grey.

The highest average annual age-adjusted pneumonia-associated hospitalisation rates were consistently seen across northern and southern Bronx, as well as pockets of northern Manhattan and Staten Island. These patterns were demonstrated for overall pneumonia-associated hospitalisation, severe pneumonia-associated hospitalisation, as well as pneumonia-associated hospitalisation stratified by setting of acquisition. While neighbourhoods with high rates were also seen in parts of Brooklyn and Queens, these higher rate neighbourhoods were less densely concentrated in comparison to the Bronx, Manhattan and Staten Island (Figs 1 and 2). Annual age-adjusted rates are shown in Supplementary Figures S1–S3.

### Semivariogram modelling

Semivariogram modelling revealed a range of spatial autocorrelation of 4859 m for overall pneumonia-associated hospitalisation and 5740 m for severe pneumonia-associated hospitalisation. CAP-associated hospitalisation had a range of spatial autocorrelation of 6005 m (Supplementary Fig. S4). We were unable to define a range of spatial autocorrelation for HCAP and HAP; therefore, cluster analyses for these subcategories were not performed. Output of the ISA analysis confirmed semivariogram modelling results.

### Cluster analyses

#### Cluster analyses for overall pneumonia-associated hospitalisation

**Fig. 3.**
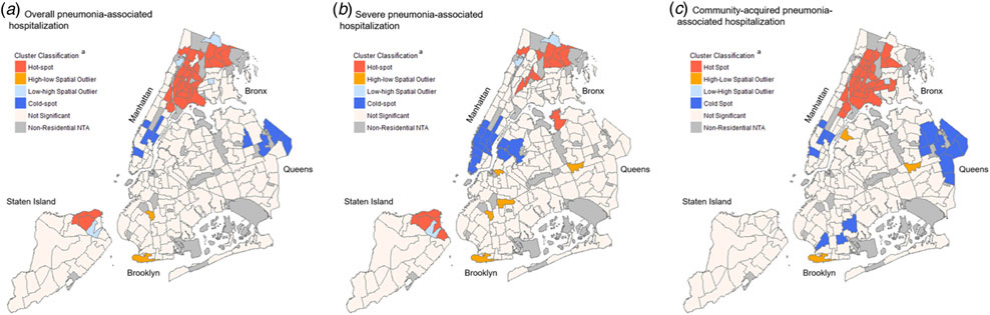
Spatial clusters of pneumonia-associated hospitalisation by Neighborhood Tabulation Area (NTA) among adults in New York City (NYC), 2010–2014. Maps showing NYC neighbourhoods according to spatial clusters of overall pneumonia-associated hospitalisation (a), severe pneumonia-associated hospitalisation (b), and community-acquired pneumonia-associated hospitalisation (c). Labels indicate the five NYC boroughs (Manhattan, Bronx, Brooklyn, Queens and Staten Island). Spatial cluster classifications were determined by local Moran's *I* cluster analyses (Supplementary Material, Methods). The analysis assigned each residential Neighborhood Tabulation Area (NTA) a cluster classification based on rates of pneumonia-associated hospitalisation (Figs 1 and 2). Non-residential NTAs were excluded from the analysis and are shown in grey. ^a^Hot spot: clusters of nearby neighbourhoods with similarly high hospitalisation rates; high–low spatial outlier: neighbourhoods with high hospitalisation rates near neighbourhoods with low hospitalisation rates; low-high spatial outlier: neighbourhoods with low hospitalisation rates near neighbourhoods with high hospitalisation rates; cold spot: clusters of nearby neighbourhoods with similarly low hospitalisation rates; not significant: neighbourhoods without statistically significant spatial clustering.

Hot spots of overall pneumonia-associated hospitalisation spanned 20 neighbourhoods in the Bronx, four in Manhattan and two in Staten Island, while cold spots were demonstrated across five neighbourhoods in lower Manhattan and four in northeastern Queens (Fig. 3a). When comparing patients who resided within hot *vs.* cold spots, we found that the greatest proportion of patients residing in hot-spot neighbourhoods identified as non-Hispanic Black (*N* = 9788; 36.8%) (Table 2). A remaining 29.4% identified as Latino/Hispanic (*N* = 7799), 19.1% as non-Hispanic other race (*N* = 5080) and 13.6% as non-Hispanic White (*N* = 3614). In contrast, the overwhelming majority of patients residing in cold spots identified as non-Hispanic White (*N* = 3113; 70.0%), while 16.4% identified as non-Hispanic other race (*N* = 730), 5.0% identified as Latino/Hispanic (*N* = 222) and 4.3% as non-Hispanic Black (*N* = 189).

**Table 2.** Comparative statistics of hot *vs.* cold spots of overall pneumonia-associated hospitalisation among adults in New York City, 2010–2014^a^

Variable	Classification	Number of hospitalisations in hot-spot neighbourhoods^b^ (%) *N* = 26 564 (100)	Number of hospitalisations in cold-spot neighbourhoods^c^ (%) *N* = 4445 (100)	*χ*^2^ ^d^	*P*-value*	Proportional difference between hot and cold spot (Δ%)^e^	95% CI^e^
Sex^f^	Male	12 459 (46.9)	2297 (51.7)	34.6	<0.01	4.8	3.2–6.4
	Female	14 104 (53.1)	2148 (48.3)	34.6	<0.01	4.8	3.2–6.4
Age group	18–24	631 (2.4)	24 (<1.0)	62.0	<0.01	1.9	1.6–2.1
	25–44	3059 (11.5)	176 (4.0)	231.9	<0.01	7.5	6.9–8.3
	45–64	9323 (35.1)	699 (15.7)	652.3	<0.01	19.4	18.1–20.6
	>65	13 551 (51.0)	3546 (79.8)	1272.3	<0.01	28.8	27.4–30.1
Setting of acquisition^g^	CAP	15 564 (58.6)	2939 (66.1)	89.4	<0.01	7.5	6.0–9.1
	HCAP	8374 (31.5)	1196 (26.9)	37.8	<0.01	4.6	3.2–6.1
	HAP	2136 (8.1)	289 (6.5)	12.3	<0.01	1.6	0.7–2.4
	VAP	490 (1.8)	21 (<1.0)	43.4	<0.01	1.3	1.1–1.6
Severity	Severe pneumonia- associated hospitalisation	11 848 (44.6)	1599 (36.0)	115.1	<0.01	8.6	7.1–10.2
	Non-severe pneumonia-associated hospitalisation	14 716 (55.4)	2846 (64.0)	115.1	<0.01	8.6	7.1–10.2
Race/ethnicity	Non-Hispanic White	3614 (13.6)	3113 (70.0)	7134.2	<0.01	56.4	55.0–57.9
	Non-Hispanic Black	9788 (36.8)	189 (4.3)	1852.2	<0.01	32.5	31.8–33.4
	Non-Hispanic Asian	221 (<1.0)	182 (4.1)	313.4	<0.01	4.0	0.8–4.1
	Latino/Hispanic	7799 (29.4)	222 (5.0)	1177.6	<0.01	24.4	23.5–25.2
	Non-Hispanic other	5080 (19.1)	730 (16.4)	18.1	<0.01	2.7	1.5–3.9
	Unknown	62 (<1.0)	9 (<1.0)	0.1	0.82	–	–
Final disposition	Discharge to home under self-care or covered skilled care	14 673 (55.2)	2860 (64.3)	128.11	<0.01	9.1	7.6–10.6
	Discharge to skilled nursing facility	6335 (23.8)	646 (14.5)	188.9	<0.01	9.3	8.1–10.5
	In-hospital death	3299 (12.4)	516 (11.6)	2.2	0.13	–	–
	Other^h^	2257 (8.6)	423 (9.6)	4.9	0.03	1.0	0.1–2.0

a Hot spots and cold spots were defined using local Moran's *I* cluster analyses, which allowed us to compare whether the hospitalisation rate of each NYC neighbourhood was significantly different from NYC as a whole and whether the hospitalisation rate of each NYC neighbourhood was significantly different from its contiguous neighbourhoods (Electronic Supplementary Material).

b Hot spot: clusters of nearby neighbourhoods with similarly high hospitalisation rates.

c Cold spot: clusters of nearby neighbourhoods with similarly low hospitalisation rates.

d Degrees of freedom for all *χ*^2^ tests were equal to 1.

e Not shown for classifications that do not have statistically significant proportional differences (*P*-value > 0.05).

f Missing sex classification for *n* = 1.

g Setting of acquisition classifications: community-acquired pneumonia (CAP); healthcare-associated pneumonia (HCAP); hospital-acquired pneumonia (HAP); ventilator-associated pneumonia (VAP).

h Other classification included patients who left against medical advice or discontinued care, or those discharged to: short-term general hospitals, facilities that provide custodial or supportive care, designated cancer centres or children's hospitals, federal healthcare facilities, hospice, inpatient rehabilitation facilities, Medicare-certified long-term care hospitals, psychiatric hospitals, critical access hospitals, or another type of healthcare institution not defined in the SPARCS code list.

*Set to 5% level of significance.

The mean age at hospitalisation for patients within hot spots of overall pneumonia-associated hospitalisation was 64 years and 76 years for patients within cold-spot neighbourhoods (*P* < 0.01).

#### Cluster analyses for severe pneumonia-associated hospitalisation

For severe pneumonia-associated hospitalisation, we found hot spots within nine neighbourhoods in northern Bronx, one neighbourhood in northern Manhattan, one in northeast Queens and three at the northern tip of Staten Island (Fig. 3b). Within hot spots of severe pneumonia-associated hospitalisation, the largest proportion of hospitalisations occurred among patients who identified as non-Hispanic Black (*N* = 2863; 35.5%), while 27.3% identified as non-Hispanic White (*N* = 2200), 22.9% identified as Latino/Hispanic (*N* = 1849) and 12.5% as non-Hispanic other race (*N* = 1011) (Table 3). The racial distribution of hospitalisations was markedly different for cold spots, with the highest proportion of patients identifying as non-Hispanic White (*N* = 4337; 56.0%) as opposed to non-Hispanic other race (*N* = 1577; 20.4%), Latino/Hispanic (*N* = 813; 10.5%) or non-Hispanic Black (*N* = 614; 7.9%).

**Table 3.** Comparative statistics of hot *vs.* cold spots of severe pneumonia-associated hospitalisation among adults in New York City, 2010–2014^a^

Variable	Classification	Number of hospitalisations in hot spot neighbourhoods^b^ (%) *N* = 8067 (100)	Number of hospitalisations in cold spot neighbourhoods^c^ (%) *N* = 7738 (100)	*χ*^2^ ^d^	*P*-value*	Proportional difference between hot and cold spot (Δ%)^e^	95% CI^e^
Sex	Male	4013 (49.7)	4214 (54.5)	35.0	<0.01	4.8	3.1–6.3
	Female	4054 (50.3)	3524 (45.5)	35.0	<0.01	4.8	3.1–6.3
Age group	18–24	90 (1.1)	63 (<1.0)	3.4	0.06	–	–
	25–44	455 (5.6)	506 (6.5)	5.4	0.02	0.9	0.1–1.7
	45–64	2194 (27.2)	1775 (22.9)	37.9	<0.01	4.3	2.9–5.6
	>65	5328 (66.1)	5394 (69.7)	24.1	<0.01	3.6	2.2–5.1
Setting of acquisition^f^	CAP	3198 (39.6)	5151 (66.6)	1147.8	<0.01	27.0	25.4–28.4
	HCAP	3349 (41.5)	2006 (25.9)	427.9	<0.01	15.6	14.1–17.1
	HAP	1094 (13.6)	548 (7.1)	177.4	<0.01	6.5	5.5–7.4
	VAP	426 (5.3)	33 (<1.0)	328.3	<0.01	4.9	4.3–5.4
Race/ethnicity	Non-Hispanic White	2200 (27.3)	4337 (56.0)	1347.3	<0.01	28.7	27.9–30.3
	Non-Hispanic Black	2863 (35.5)	614 (7.9)	1746.0	<0.01	27.6	26.3–28.8
	Non-Hispanic Asian	127 (1.6)	376 (4.9)	137.2	<0.01	3.3	2.7–3.8
	Latino/Hispanic	1849 (22.9)	813 (10.5)	433.7	<0.01	12.4	11.3–13.6
	Non-Hispanic other	1011 (12.5)	1577 (20.4)	177.1	<0.01	7.9	6.7–9.0
	Unknown	17 (<1.0)	21 (<1.0)	0.4	0.54	–	–
Final disposition	Discharge to home under self-care or covered skilled care	1581 (19.6)	5118 (66.1)	3501.6	<0.01	46.5	45.2–47.9
	Discharge to skilled nursing facility	3910 (48.5)	1003 (13.0)	2322.7	<0.01	35.5	34.2–36.8
	In-hospital death	1976 (24.5)	780 (10.1)	569.0	<0.01	14.4	13.2–15.6
	Other^g^	600 (7.4)	837 (10.8)	54.2	<0.01	3.4	2.5–4.3

a Hot spots and cold spots were defined using local Moran's *I* cluster analyses, which allowed us to compare whether the hospitalisation rate of each NYC neighbourhood was significantly different from NYC as a whole and whether the hospitalisation rate of each NYC neighbourhood was significantly different from its contiguous neighbourhoods (Electronic Supplementary Material).

b Hot spot: clusters of nearby neighbourhoods with similarly high hospitalisation rates.

c Cold spot: clusters of nearby neighbourhoods with similarly low hospitalisation rates.

d Degrees of freedom for all *χ*^2^ tests were equal to 1.

e Not shown for classifications that do not have statistically significant proportional differences (*P*-value > 0.05).

f Setting of acquisition classifications: community-acquired pneumonia (CAP); healthcare-associated pneumonia (HCAP); hospital-acquired pneumonia (HAP); ventilator-associated pneumonia (VAP).

g Other classification included patients who left against medical advice or discontinued care, or those discharged to: short-term general hospitals, facilities that provide custodial or supportive care, designated cancer centres or children's hospitals, federal healthcare facilities, hospice, inpatient rehabilitation facilities, Medicare-certified long-term care hospitals, psychiatric hospitals, critical access hospitals, or another type of healthcare institution not defined in the SPARCS code list.

*Set to 5% level of significance.

The mean age at hospitalisation for patients within hot spots of severe pneumonia-associated hospitalisation was 64 years and 72 years for patients within cold-spot neighbourhoods (*P* < 0.01). For hospitalisations among hot-spot patients, the median LOS was 10.0 days (IQR: 6.0–18.0) compared with 5.0 days (IQR: 3.0–10.0) for cold-spot patients. Only 19.6% (*N* = 1581) of patients residing in hot spots were released to their home under self-care or covered skilled care; however, this is where the greatest proportion of cold-spot patients were discharged (*N* = 5118; 66.1%) (*P* < 0.01). In contrast, the largest proportion of patients residing in hot spots were released to skilled nursing facilities (*N* = 3910; 48.5%); this proportion was significantly less for cold-spot patients (*N* = 1003; 13.0%) (*P* < 0.01). Nearly one-quarter of patients within hot spots experienced in-hospital death (*N* = 1976; 24.5%), whereas approximately one-tenth of cold-spot patients experienced in-hospital death (*N* = 780; 10.1%) (*P* < 0.01).

#### Cluster analyses for CAP-associated hospitalisation

For CAP-associated hospitalisation, hot spots were revealed for 26 neighbourhoods across Bronx and northern Manhattan (Fig. 3c). Cold spots were observed in northeast Queens, as well as seven neighbourhoods in southern Manhattan and Brooklyn.

The mean age at hospitalisation for patients within hot spots of CAP-associated hospitalisation was 63 years, as opposed to 72 years for cold spots (*P* < 0.01). Once more, the largest proportion of patients residing in hot spots identified as non-Hispanic Black (*N* = 9063; 35.2%), with approximately one-third of individuals identifying as Latino/Hispanic (*N* = 8535; 33.2%) (Table 4). For hospitalisations among hot-spot patients, the median LOS was 5.0 days (IQR: 3.0–10.0) compared with 6.0 days (IQR: 3.0–10.0) for cold-spot patients. In examining the severity of CAP-associated hospitalisations, proportions were similar for hot spots *vs.* cold spots (*P* > 0.05). We did not identify statistically significant differences in the proportion of patients discharged to the home under self-care or covered skilled care for CAP-associated hospitalisation clusters, and the proportion of patients who experienced in-hospital death was only slightly higher for hot spots (*N* = 2967; 11.5%) compared with cold spots (*N* = 780; 10.7%) (*P* = 0.05). However, a larger percentage of patients were discharged to skilled nursing facilities within hot spots (*N* = 4968; 19.3%) compared with cold spots (*N* = 1187; 16.2%) (*P* < 0.01).

**Table 4.** Comparative statistics of hot *vs.* cold spots of community-acquired pneumonia-associated hospitalisation among adults in New York City, 2010–2014^a^

Variable	Classification	Number of hospitalisations in hot spot neighbourhoods^b^ (%) *N* = 25 736 (100)	Number of hospitalisations in cold spot neighbourhoods^c^ (%) 7305 (100)	*χ*^2^ ^d^	*P*-value*	Proportional difference between hot and cold spot (Δ%)^e^	95% CI^e^
Sex^f^	Male	12 051 (46.8)	3684 (50.4)	29.5	<0.01	3.6	2.3–4.9
	Female	13 684 (53.2)	3621 (49.6)	29.5	<0.01	3.6	2.4–4.9
Age group	18–24	688 (2.7)	90 (1.2)	50.8	<0.01	1.5	1.1–1.8
	25–44	3220 (12.5)	444 (6.1)	238.2	<0.01	6.4	5.7–7.1
	45–64	9392 (36.5)	1613 (22.1)	531.5	<0.01	14.4	13.3–15.5
	>65	12 436 (48.3)	5158 (70.6)	1490.4	<0.01	22.3	21.1–23.5
Race/ethnicity	Non-Hispanic White	2411 (9.4)	4106 (56.2)	7881.3	<0.01	46.8	45.6–48.0
	Non-Hispanic Black	9063 (35.2)	1218 (16.7)	911.8	<0.01	18.5	17.5–19.6
	Non-Hispanic Asian	249 (<1.0)	488 (6.7)	848.9	<0.01	5.7	5.1–6.3
	Latino/Hispanic	8535 (33.2)	436 (6.0)	2126.2	<0.01	27.2	26.4–28.0
	Non-Hispanic other	5420 (21.1)	1047 (14.3)	163.2	<0.01	6.8	5.8–7.7
	Unknown	58 (<1.0)	10 (<1.0)	1.8	0.18	–	–
Severity	Severe pneumonia-associated hospitalisation	10 722 (41.7)	2992 (41.0)	1.1	0.29	–	–
	Non-severe pneumonia-associated hospitalisation	15 014 (58.3)	4313 (59.0)	1.1	0.29	–	–
Final disposition	Discharge to home under self-care or covered skilled care	15 556 (60.5)	4506 (61.7)	3.6	0.06	–	–
	Discharge to skilled nursing facility	4968 (19.3)	1187 (16.2)	34.8	<0.01	3.1	2.1–4.0
	In-hospital death	2967 (11.5)	780 (10.7)	4.0	0.05	–	–
	Other^g^	2245 (8.7)	832 (11.4)	47.6	<0.01	2.7	1.9–3.5

a Hot spots and cold spots were defined using local Moran's *I* cluster analyses, which allowed us to compare whether the hospitalisation rate of each NYC neighbourhood was significantly different from NYC as a whole and whether the hospitalisation rate of each NYC neighbourhood was significantly different from its contiguous neighbourhoods (Electronic Supplementary Material).

b Hot spot: clusters of nearby neighbourhoods with similarly high hospitalisation rates.

c Cold spot: clusters of nearby neighbourhoods with similarly low hospitalisation rates.

d Degrees of freedom for all *χ*^2^ tests were equal to 1.

e Not shown for classifications that do not have statistically significant proportional differences (*P*-value > 0.05).

f Missing sex classification for *n* = 1.

g Other classification included patients who left against medical advice or discontinued care, or those discharged to: short-term general hospitals, facilities that provide custodial or supportive care, designated cancer centres or children's hospitals, federal healthcare facilities, hospice, inpatient rehabilitation facilities, Medicare-certified long-term care hospitals, psychiatric hospitals, critical access hospitals or another type of healthcare institution not defined in the SPARCS code list.

*Set to 5% level of significance.

## Discussion

We found distinct spatial patterns in the rates of overall pneumonia-associated hospitalisation, severe pneumonia-associated hospitalisation and CAP-associated hospitalisation for NYC during 2010–2014. In applying a spatial framework to investigate the epidemiology of pneumonia, we incorporated the inter-relatedness of race/ethnicity, health behaviours and health services in representing rates of pneumonia-associated hospitalisation [18].

To reduce illness and death from pneumonia, it is important to understand where burden is greatest and the setting in which NYC residents acquire infection. Our previous study found that 60% of pneumonia-associated hospitalisations are attributable to CAP, and here we identified neighbourhoods for potential interventions [4]. Specific risk factors for each neighbourhood cannot be ascertained without regression modelling. However, patterns in the spatial clusters for CAP largely reflected segregation by race/ethnicity that exists across the city and inequities in poverty and chronic disease, as described below. Setting of acquisition results differed from estimates presented by Corrado *et al*. due to variations in the definition of pneumonia-associated hospitalisation. Corrado *et al*. present pneumonia-associated hospitalisations based on diagnostic codes among any of the discharge diagnoses, while we include only those based on principal diagnosis [4].

Throughout this study, we demonstrated that neighbourhoods in the Bronx and northern Staten Island were disproportionately affected by high rates of pneumonia-associated hospitalisation. Twenty of the 36 NTAs that make up the Bronx were classified as hot spots of overall pneumonia-associated hospitalisation, revealing a high burden across the borough. For Staten Island, only a small locality was defined as a hot spot of overall pneumonia-associated hospitalisation and severe pneumonia-associated hospitalisation. These communities have higher Latino/Hispanic and non-Hispanic Black populations compared with other neighbourhoods within this city. In the Bronx, 45% of individuals identify as being of White race, while approximately 64% of residents in Manhattan identify as White [10]. For neighbourhoods within the Staten Island hot spot, 39% of individuals identify as White, while remaining portions of the borough range between 70% and 85% White [19]. Area-based poverty may be one contributing factor to pneumonia-risk within NYC. Approximately 31% of individuals in the Bronx live below the Federal Poverty Level (FPL), representing the highest percentage among the five boroughs, and 20% live below the FPL in the Staten Island hot spot, the highest percentage in that borough [19, 20].

Additionally, increased risk of pneumonia among individuals with chronic conditions such as asthma and diabetes has been described [21, 22]. To this end, the Bronx has rates of adult hospitalisation for diabetes and asthma that are approximately twice the citywide rate [20]. Adults within the Staten Island hot spot also have higher rates of diabetes and asthma hospitalisations compared with the borough and city overall [19]. Finally, the rate of premature death (death before age of 65 years) in certain neighbourhoods of the Bronx is over four times the rate of premature death in higher socioeconomic status neighbourhoods of southern Manhattan [20]. These associations reinforce that efforts to reduce pneumonia morbidity and mortality cannot be dissociated from broader health equity interventions. Instead, pneumonia prevention would benefit from being conducted in conjunction with efforts to address underlying social conditions that make residents of these hot spots more vulnerable to infectious and chronic disease as well as premature death [23, 24].

Cold spots of pneumonia-associated hospitalisation were densely concentrated in southern Manhattan and western Brooklyn. The average age of patients experiencing a severe pneumonia-associated hospitalisation was eight years lower within hot spots *vs.* cold spots. Given the high proportion of poverty in the Bronx and Staten Island, we hypothesise that premature age at hospitalisation is a downstream effect of disparities in access to care due to factors such as race, income inequality and access to health care [25–27]. Between 2010 and 2015, which overlaps the study period, there was a 4.1% increase in comprehensive health insurance coverage among NYC residents [28]. Additionally, in 2015, NYC launched an effort to expand community health centres in 25 underserved neighbourhoods to build primary care capacity [29]. As data become available, our analyses can be replicated to understand if and how efforts to improve community health have impacted rates and clusters of severe pneumonia-associated hospitalisation. Furthermore, our results established that the LOS for patients admitted for severe pneumonia was longer and the proportion of patients who experienced in-hospital death was over two times higher for residents of hot spots *vs.* cold spots. Therefore, directing prevention and treatment efforts towards severe pneumonia-associated hospitalisation hot spots creates a potential opportunity to more substantially impact pneumonia-associated mortality.

### Limitations

While this study adds to our understanding of the spatial epidemiology of pneumonia-associated hospitalisations within NYC, several limitations exist. Foremost, the quality of data for classifying pneumonia within SPARCS is unknown. ICD-9-CM coding is subject to differential facility management and medical charting practices, with the potential for inaccuracies [30, 31]. We assumed that the use of respiratory failure or sepsis as a principal diagnosis represented a severe pneumonia-associated hospitalisation, but have not verified this through chart review. Additionally, hospitals may be incentivised to code and bill for diagnoses that maximise insurance reimbursement [31]. We controlled for this bias, in part, by utilizing hospitalisation definitions set forth by Lindenauer *et al*. [9] The accuracy of patient-level records in SPARCS is unknown, particularly with respect to race/ethnicity and residential information. For address data, precise reporting is a challenge for individuals of low socioeconomic status subject to housing instability, and this study does not take into account persons who reside, either short-term or long-term, within non-residential NTAs [32]. Furthermore, it is important to distinguish that SPARCS uniquely identifies hospitalisations for which pneumonia has been listed as a discharge diagnosis, and not pneumonia-confirmed cases. Despite this, rates derived from SPARCS likely underestimate the incidence of pneumonia in NYC during 2010–2014, as we did not account for outpatient pneumonia cases. Limitations associated with the defined setting of acquisition are described in detail by Corrado *et al*. [4] Due to the large numbers of hospitalisations within each hot and cold spot, the proportional differences between the clusters are overwhelmingly statistically significant at a 5% level of significance. Results should therefore be interpreted with respect to clinical rather than statistical significance [33].

This study is based on average-annual age-adjusted rates for 2010–2014, without accounting for inter-annual variability in hospitalisations during this period. While exploring the drivers of this variation (e.g. seasonal and pandemic influenza) falls out of the scope of this study, annual rates are presented in Supplementary Figures S1–S3. Finally, results of the comparative analyses between hot and cold spots of overall, severe and CAP-associated hospitalisations may reflect confounding demographic and clinical factors. This limitation will be explored by developing future multivariable regression models. Based on the spatial autocorrelation of pneumonia-associated hospitalisation rates detected in this study, a geographically weighted regression would likely be the most suitable course of action. Given the constraints of SPARCS data described in this section, further research is required to identify covariates of CAP and HAP in NYC at the appropriate spatial scale.

This study was built off of previous DOHMH examinations of the setting of acquisition, hospitalisation rates and mortality rates associated with pneumonia in NYC [4]. This study helps to reveal how pneumonia-associated hospitalisations vary within and between NYC boroughs. Additionally, our analyses detail geographic differences in the clinical and demographic characterisations of pneumonia-associated hospitalisations.

Reducing pneumonia mortality within NYC will likely require a systems approach, leveraging a combination of citywide initiatives and community engagement activities to raise health equity [34]. Certain strategies, such as improving standards for inpatient care can be addressed through governmental and hospital policy. However, efforts to target risk factors for pneumonia within the community must incorporate the specific needs, culture and infrastructure of NYC neighbourhoods. Based on results of this study, we recommend that additional research be focused on understanding the reasons for higher rates in hot spots through geographic regression modelling, and heterogeneities in clinical management of pneumonia across NYC be examined through facility-level analyses. We propose that resources intended for the improvement of pneumonia morbidity and mortality be directed towards neighbourhoods within the Bronx, as well as northern Manhattan and Staten Island.

## Disclaimer

The findings and conclusions in this article are those of the authors and do not necessarily represent the official position of NYC DOHMH or CDC.

## Supplementary material

For supplementary material accompanying this paper visit https://doi.org/10.1017/S0950268818003060. click here to view supplementary material
