# Supplementary material for: Geospatial cluster analyses of pneumonia-associated hospitalisations among adults in New York City, 2010–2014
Source: Epidemiol Infect. 2018 Nov 19;147:e51. doi: 10.1017/S0950268818003060 (PMC6518844; doi:10.1017/S0950268818003060)
Supplement: Supplementary file 1 [file S0950268818003060sup001.docx]

**Epidemiology and Infection**

Geospatial cluster analyses of pneumonia-associated hospitalizations among adults in New York City, 2010–2014

P.A. KACHE, T. JULIEN, R.E. CORRADO, N.M. VORA, D. DASKALAKIS, J.K. VARMA, D.E. LUCERO

Supplementary Material

Methods

*Geostatistical Analyses*

The empirical semivariogram plotted semivariance *γ(h)*, using Equation 1, where *(h)* is the lag or Euclidian distance between a pair of points, and *z* is the value of a parameter at points *x_i_* and *x_j_*. We defined *(h)* as the lag, in meters, between the centroids of NTAs *x_i_* and *x_j_*_,_ and *z* as the hospitalization rates for NTAs *x_i_* and *x_j_.* [14]

**(1)** $\gamma\left( h \right)=\frac{1}{2}\left[ z\left( x_{i} \right)-z\left( x_{j} \right) \right]^{2}$

To summarize the variation of a parameter in space, we “binned” the data of the empirical semivariogram using experimental semivariance *γ_2_*(*h*) (Equation 2). Here *N (h)* is the number of pairs separated by lag *(h)*, and *z* is the value of a parameter at points *x_i_* and *x_i_+h*. Specifically, *N(h)* represents the number of pairwise NTAs separated by lag *(h)* and *z* denotes the hospitalization rates for NTAs *x_i_* and *x_i_+h*. [14]

**(2)** $\gamma_{2}\left( h \right)=\frac{1}{2N(h)}\sum_{i=1}^{N(h)} \left[ z\left( x_{i} \right)-z\left( x_{i}+h \right) \right]^{2}$

Finally, a statistical model was fit through the experimental semivariogram to describe three output parameters: (1) the nugget (*c_0_*), which is the semivariance for *(h)* = 0 (i.e., measurement error that is not spatially dependent); (2) the sill (*c*), which is the value of semivariance at which the semivariogram becomes asymptotic; and (3) the range of spatial autocorrelation (*a*), which is the value of *(h)* at which the semivariogram becomes asymptotic, and the distance beyond which the parameter is no longer correlated. We fit the models using an exponential function (Equation 3). [14]

**(3)** $\gamma_{3}\left( h \right)=c_{0}+c[1-\exp\left( -\frac{|h|}{a} \right)]$

*Cluster Analyses*

Local Moran’s *I* allowed us to compare whether the hospitalization rate of each NYC neighborhood was significantly different from NYC as a whole and whether the hospitalization rate of each NYC neighborhood was significantly different from its contiguous neighborhoods. Analyses derived a local Moran’s *I* value for each of the 188 residential NTAs using Equation 4. [16] In this computation, pairs of NTAs are represented by *i* and *j*, where *z(x_i_)* and *z(x_j_)* are the hospitalization rates for the two NTAs being compared. The average hospitalization rate for NYC is shown by $\bar{x}$, *N* denotes the number of *j* NTAs being compared to NTA *i (*i.e., 188-1=187), and *w_ij_* is an assigned weight dependent on the distance between NTAs *i* and *j*. The weight is informed by the range of spatial autocorrelation defined through semivariogram modeling, such that pairs of NTAs within the range are designated a weight of one. For distances that exceed the range of spatial autocorrelation, the weight is set equivalent to zero.

**(4)** $I_{i}={z(x}_{i})-\bar{x}\sum_{j=1}^{N} w_{ij}{z(x}_{j})-\bar{x}$


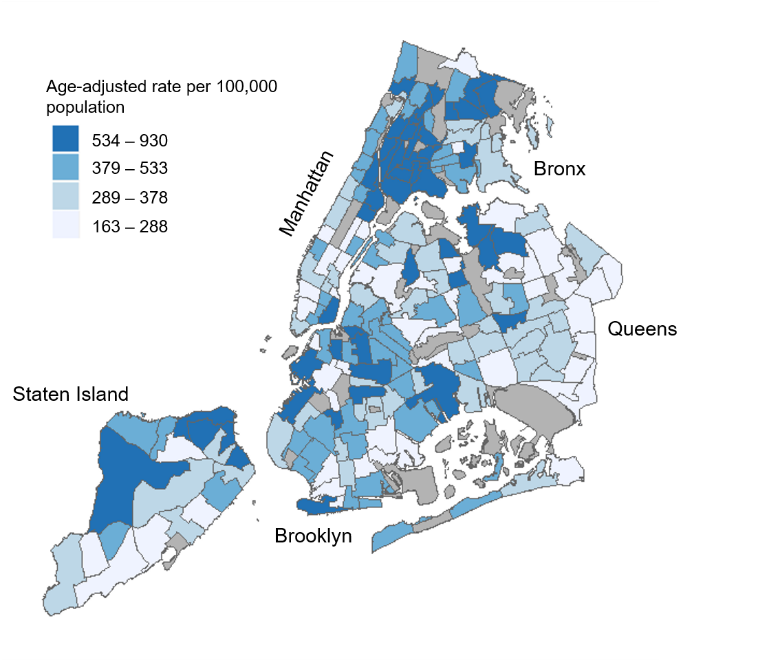


**Supplementary Figure S1a.** 2010


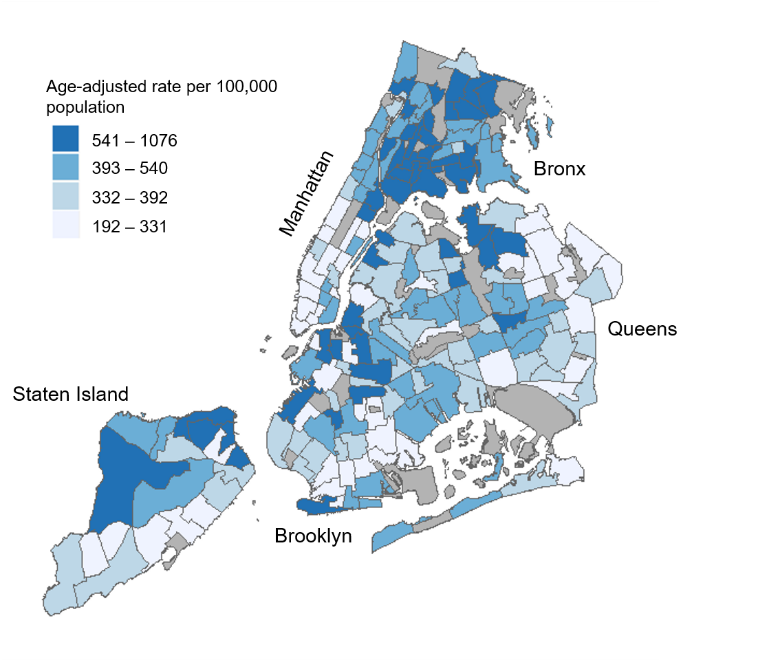


**Supplementary Figure S1b.** 2011


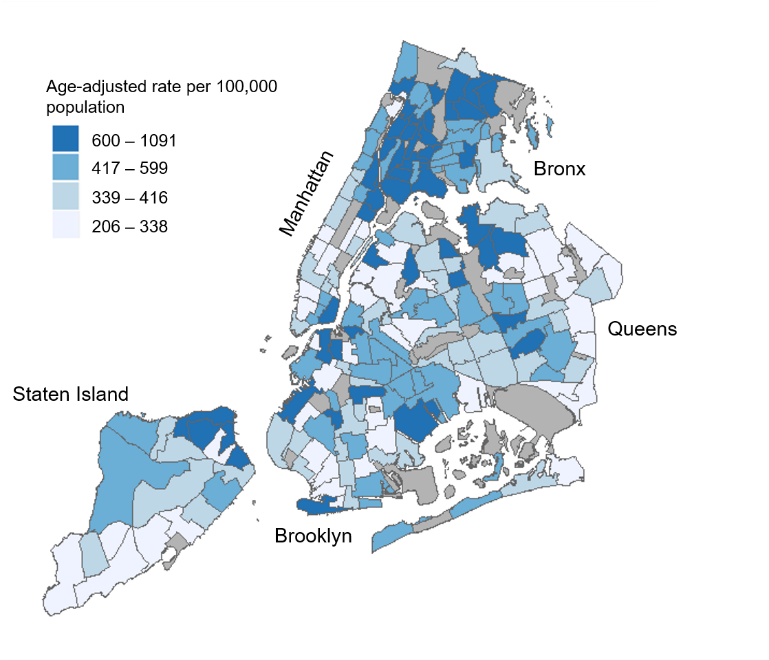


**Supplementary Figure S1c.** 2012


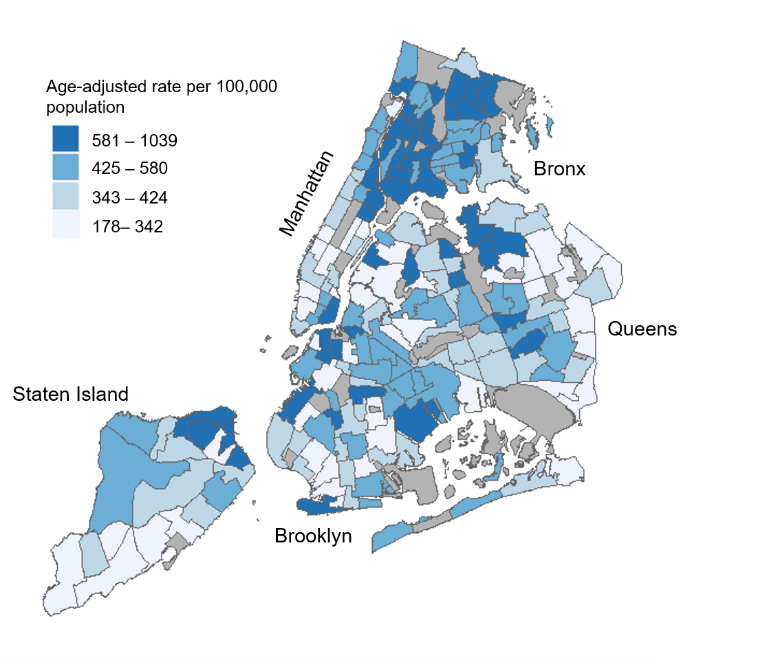


**Supplementary Figure S1d.** 2013


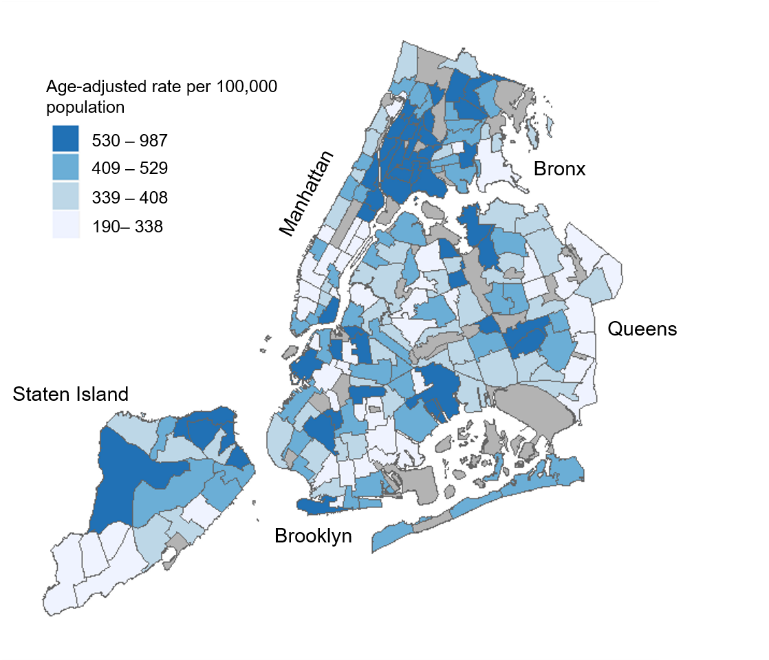


**Supplementary Figure S1e.** 2014

**Supplementary Figure S1.** Annual age-adjusted pneumonia-associated hospitalization rates of overall pneumonia-associated hospitalization among adults in New York City, 2010–2014

**Supplementary Figure S1.** Maps showing New York City (NYC) neighborhoods according with annual age-adjusted hospitalization rates of overall pneumonia-associated hospitalization among adults, 2010 – 2014. Labels indicate the five NYC boroughs (Manhattan, Bronx, Brooklyn, Queens, and Staten Island). Hospitalization rates were calculated for each residential Neighborhood Tabulation Area (NTA) based on hospital discharge data from the New York Statewide Planning and Research Cooperative System, and are divided into quartile classifications, with an equal number of residential NTAs in each class. Higher hospitalization rates are shown in darker blue, and lower hospitalization rates shown in lighter blue. Non-residential NTAs were excluded from the analysis and are shown in grey.


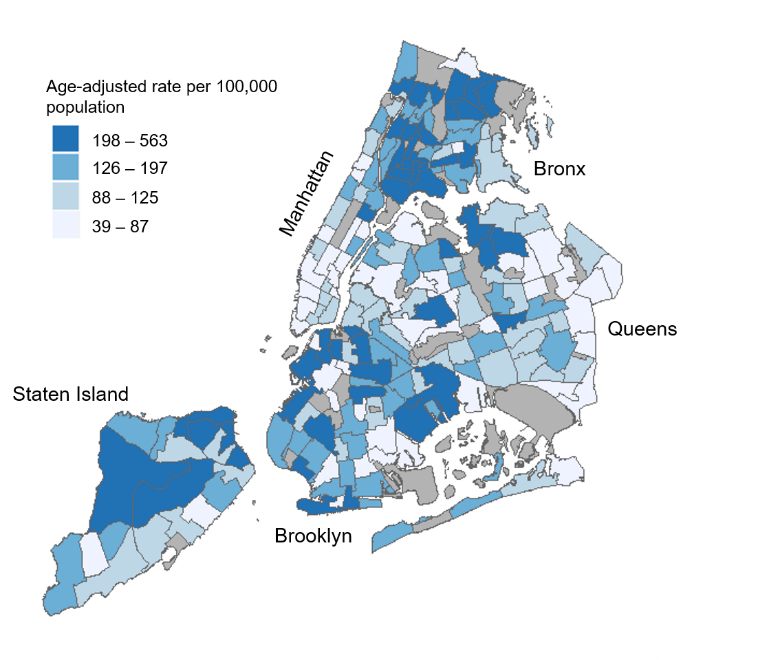


**Supplementary Figure S2a.** 2010


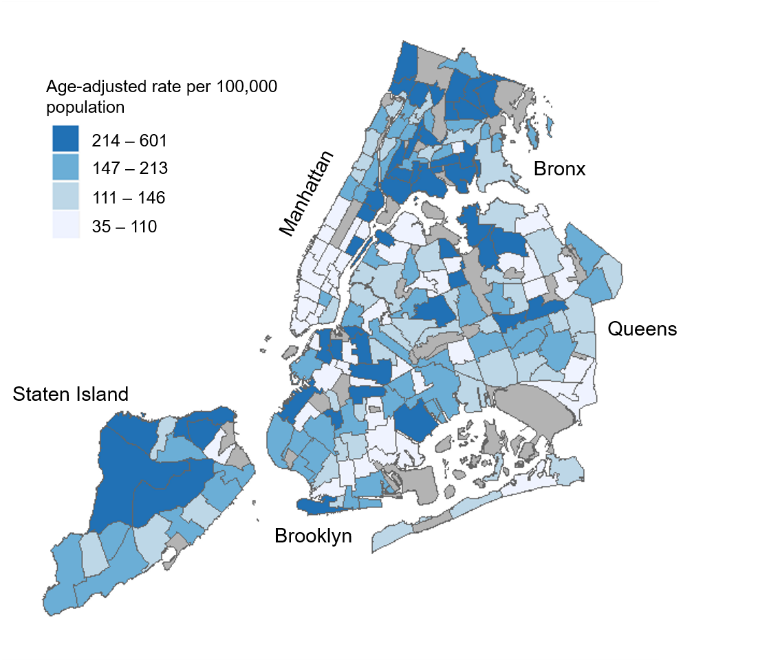


**Supplementary Figure S2b.** 2011


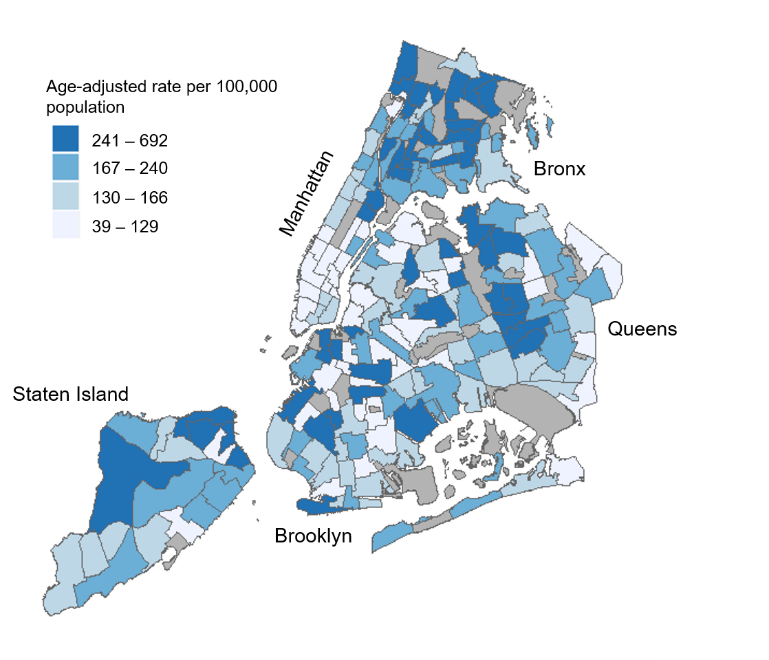


**Supplementary Figure S2c.** 2012


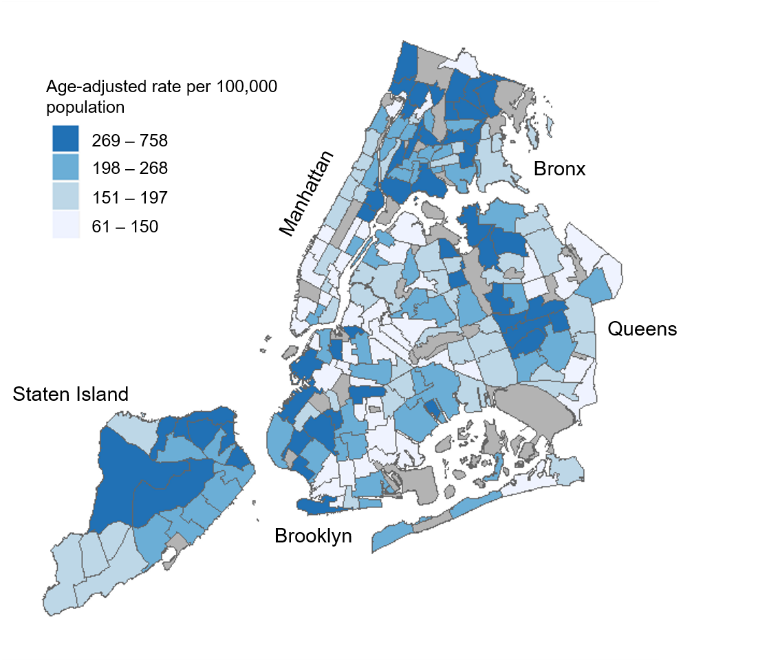


**Supplementary Figure S2d.** 2013


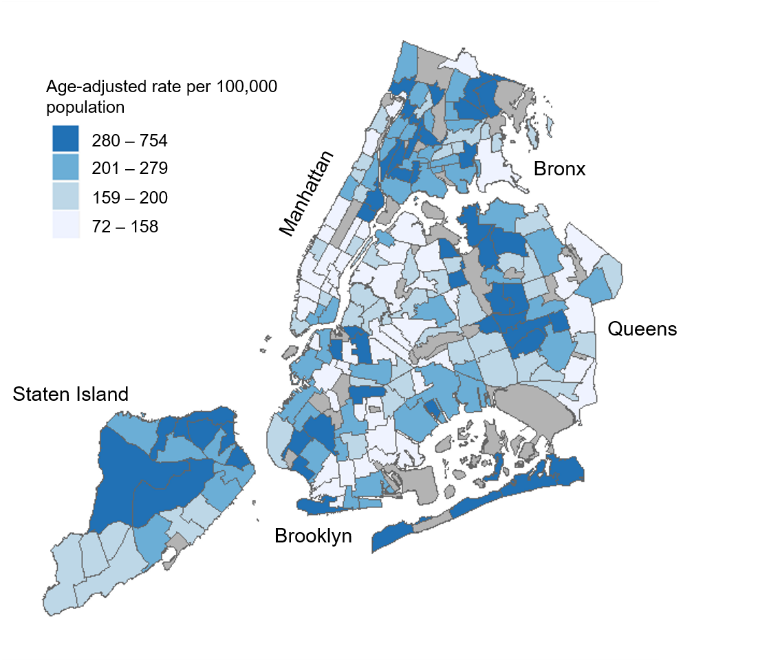


**Supplementary Figure S2e.** 2014

**Supplementary Figure S2.** Annual age-adjusted pneumonia-associated hospitalization rates of severe pneumonia-associated hospitalization among adults in New York City, 2010–2014

**Supplementary Figure S2.** Maps showing New York City (NYC) neighborhoods according with annual age-adjusted hospitalization rates of severe pneumonia-associated hospitalization among adults, 2010 – 2014. Labels indicate the five NYC boroughs (Manhattan, Bronx, Brooklyn, Queens, and Staten Island). Hospitalization rates were calculated for each residential Neighborhood Tabulation Area (NTA) based on hospital discharge data from the New York Statewide Planning and Research Cooperative System, and are divided into quartile classifications, with an equal number of residential NTAs in each class. Higher hospitalization rates are shown in darker blue, and lower hospitalization rates shown in lighter blue. Non-residential NTAs were excluded from the analysis and are shown in grey.


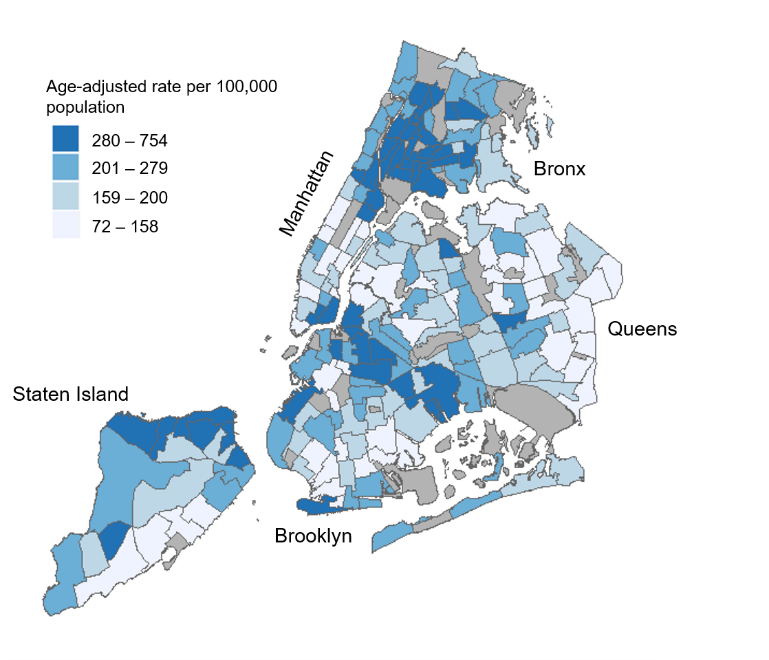


**Supplementary Figure S3a.** 2010


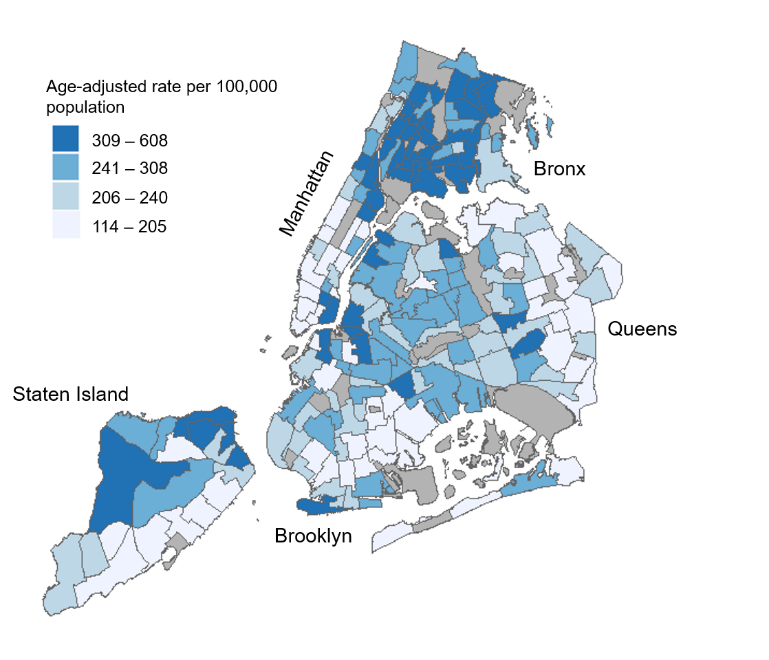


**Supplementary Figure S3b.** 2011


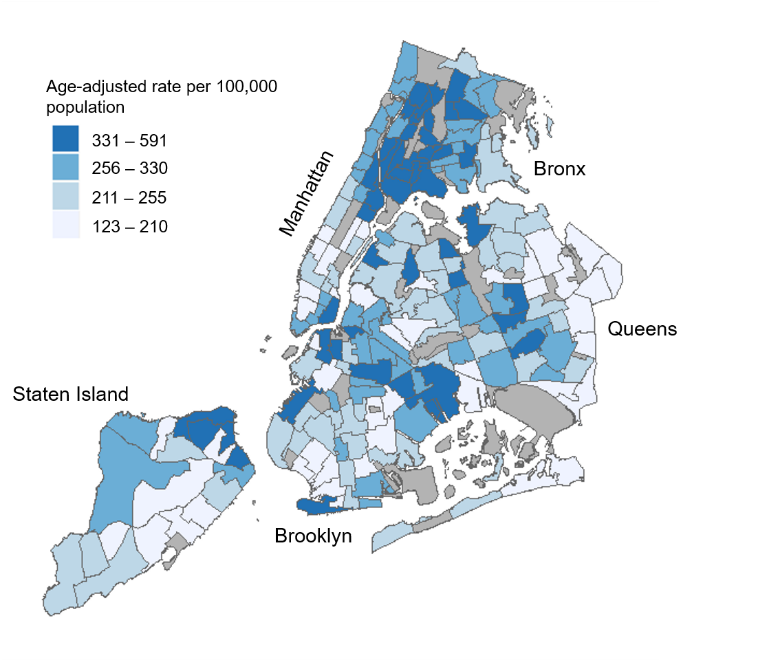


**Supplementary Figure S3c.** 2012


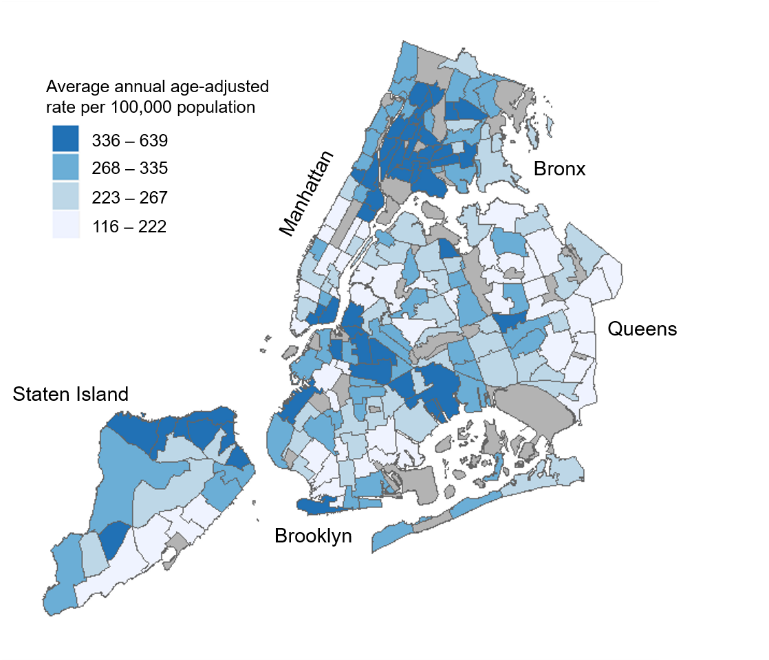


**Supplementary Figure S3d.** 2013


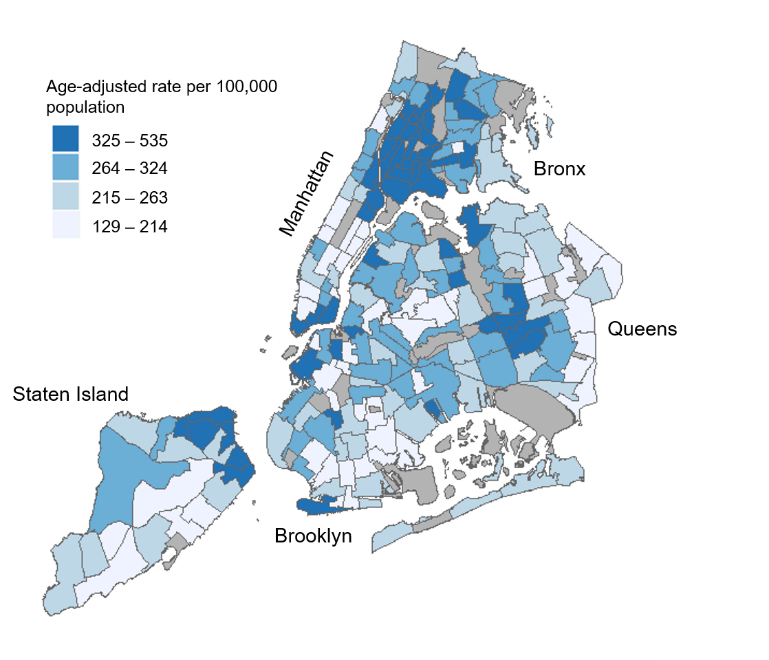


**Supplementary Figure S3e.** 2014

**Supplementary Figure S3.** Annual age-adjusted pneumonia-associated hospitalization rates of community-acquired pneumonia-associated hospitalization among adults in New York City, 2010–2014

**Supplementary Figure S3.** Maps showing New York City (NYC) neighborhoods according with annual age-adjusted hospitalization rates of community-acquired pneumonia-associated hospitalization among adults, 2010 – 2014. Labels indicate the five NYC boroughs (Manhattan, Bronx, Brooklyn, Queens, and Staten Island). Hospitalization rates were calculated for each residential Neighborhood Tabulation Area (NTA) based on hospital discharge data from the New York Statewide Planning and Research Cooperative System, and are divided into quartile classifications, with an equal number of residential NTAs in each class. Higher hospitalization rates are shown in darker blue, and lower hospitalization rates shown in lighter blue. Non-residential NTAs were excluded from the analysis and are shown in grey.

**Supplementary Figure S4.** Exponential semivariogram models of pneumonia-associated hospitalization among adults in New York City, 2010–2014

**Supplementary Figure S4a.** Overall pneumonia-associated hospitalization

**Supplementary Figure S4b.** Severe pneumonia-associated hospitalization


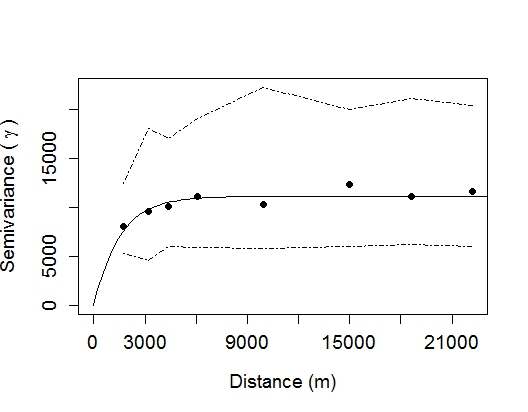

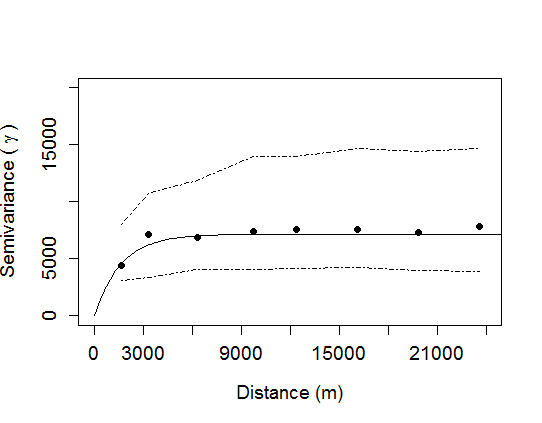

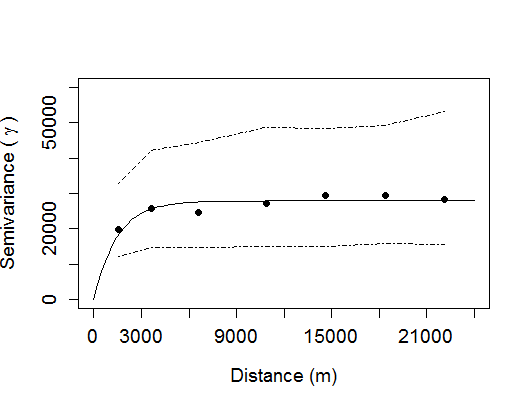


**Supplementary Figure S4c.** Community-acquired pneumonia-associated hospitalization

**Supplementary Figure S4.** Exponential semivariograms model how neighborhood-level pneumonia-associated hospitalization rates vary across New York City as a function of the distance between the neighborhoods being compared. The x-axis shows the distance (i.e., lag) between the centroids of pairwise Neighborhood Tabulation Areas (NTAs) being compared. The y-axis shows the semivariance (γ) of NTA-level hospitalization rates for overall pneumonia-associated hospitalization (Figure S4a), severe pneumonia-associated hospitalization (Figure S4b), and community-acquired pneumonia-associated hospitalization (Figure S4c) at different scales. The solid circles represent the binned semivariance of thousands of uniquely paired NTAs. The solid line represents a fitted model using an exponential function, with the distance at which the function becomes asymptotic indicating the range of spatial autocorrelation. The dashed lines depict a confidence band for the fitted model, generated from 9,999 simulations (Supplementary Material).
